# Circulating tumor cells: silent predictors of metastasis

**DOI:** 10.12688/f1000research.11313.1

**Published:** 2017-08-14

**Authors:** LanLan Zhou, David T. Dicker, Elizabeth Matthew, Wafik S. El-Deiry, R. Katherine Alpaugh

**Affiliations:** 1 Fox Chase Cancer Center, Philadelphia, PA, USA

**Keywords:** CTC, Circulating Tumour Cells, Isolation Technique, Immunophenotyping, Oncology

## Abstract

Circulating tumor cells (CTCs) were added to the arsenal of clinical testing in 2004 for three cancer types: metastatic breast, prostate, and colorectal cancer. CTCs were found to be an independent prognostic indicator of survival for these three diseases. Multiple enrichment/isolation strategies have been developed and numerous assay applications have been performed using both single and pooled captured/enriched CTCs. We have reviewed the isolation techniques and touched on many analyses. The true utility of a CTC is that it acts as a “silent” predictor of metastatic disease. The mere presence of a single CTC is an indication that disease has spread from the primary site. Comments and suggestions have been set forth for CTCs and cell-free DNA to be used as a screening panel for the early detection of disease recurrence and metastatic spread, providing the opportunity for early intervention with curative intent to treat metastatic disease.

## Introduction

The description of circulating tumor cells (CTCs) in the peripheral blood of cancer patients dates back to Ashworth’s observations in 1869 ^1^. The isolation and detection of CTCs in the blood has proven to be technically challenging. CTCs are rare compared to other circulating cells ^2^, and there is a lack of unique, well-defined universal surface targets amongst all malignant cell types. To complicate the issue, there is the possibility of a change in surface targets dependent on CTC maturation status, e.g. during epithelial to mesenchymal transition (EMT) ^3^. In spite of these challenges, researchers have developed a variety of techniques to capture and identify these cells. CellSearch ^TM^ (Menarini, Bologna, Italy) is generally thought of as the “gold standard” and remains the only analytically valid and FDA-cleared platform for prognostic use in breast, prostate, and colorectal cancers. Studies by Allard, Cristofanilli, de Bono, and Cohen established enumeration of CTCs as an independent prognostic factor for survival ^4– 11^. In addition, it has been shown that serial evaluations of CTCs during treatments show fluctuations and can serve as a biomarker of response to monitor therapies ^12– 16^. However, even though physicians do not treat patients based on the presence of CTCs, there is utility in these cells. CTCs have been labeled as a “liquid biopsy”, a source of tumor cells from the blood when conventional tissue biopsies are not attainable, which can be employed to phenotypically characterize the tumor and as a DNA/RNA source for genomic interrogation.

We will briefly summarize examples of various techniques to obtain CTCs and then review the analyses of CTCs. In conclusion, we will comment on CTCs as a “silent predictor”.

## Isolation techniques

Isolation strategies can fall into two broad categories: 1) immuno-based capture/depletion that relies on immunological recognition of unique biomarkers (i.e. EpCAM) and 2) techniques that exploit a physical property of the CTCs. Although these various techniques are utilized for the enrichment of CTCs, the classification of the captured cells as epithelial cancer cells relies on immuno-staining to confirm whether the isolated cells are tumor or normal circulating cellular components.

### Immuno-based capture/depletion for enrichment and/or immuno-labeling for identification

CellSearch ^TM^ enriches for CTCs in whole blood by first labeling with an avidin-biotin anti-EpCAM-ferrofluid complex followed by magnetic capture. CTCs are then differentially stained using DAPI to identify nucleated cells, epithelial structural cytokeratins (CK8, CK18, and CK19), and anti-CD45 to differentiate CTCs from circulating white blood cells (WBCs).

The LiquidBiopsy platform (Cynvenio Biosystems, Westlake Village, CA, USA) enriches for CTCs by initially differentially immuno-staining with a DAPI, CK, anti-CD45, anti-EpCAM-biotin cocktail and then introduces avidin-ferrofluid for magnetic immobilization. CTCs with a CTC/WBC ratio of 1% or more are analyzed with next-gene sequencing (NGS). The LiquidBiopsy platform isolates circulating cell-free DNA (ccfDNA) from the plasma, DNA from whole blood to evaluate somatic mutations, and CTC-derived DNA (ctcDNA) for each sample ^17^.

Several other techniques utilize flow microchips with immobilized EpCAM antibody either on micro-posts ^16^ or in a “herringbone” design ^18^.

The Epic system (Epic Sciences Inc., San Diego, CA, USA) has no enrichment step other than red blood cell (RBC) lysis. All nucleated cells are deposited on glass slides, which are then differentially immuno-stained, enabling CTC identification ^19^.

The AdnaTest (AdnaGen AG, Langenhagen, Germany) uses anti-EpCAM-labeled magnetic beads for CTC capture. CTCs are lysed to yield mRNA, then analyzed by RT-PCR for tumor-associated expression patterns. CTCs are not enumerated ^20^.

Saucedo-Zeni *et al*. have demonstrated the use of a medical wire functionalized with EpCAM antibodies and placed within a peripheral vein to capture CTCs *in vivo* ^21^.

### Physical property-based enrichment

Researchers have taken advantage of the larger size and/or more structurally rigid properties of CTCs to enrich samples. Among these are CellSieve (Creatv MicroTech Inc., Potomac, MD, USA) ^22, 23^ and ISET (RareCells Diagnostics, Paris, France) ^24^, which use a filter-based membrane with a specific pore size.

Parsortix (ANGLE plc, Guildford, UK) ^25^ and FMSA (Pennsylvania State University, Department of Biomedical Engineering, State College, PA, USA) ^26^ microfluidic devices not only use size but also depend on the ability of the CTCs to deform in their enrichment strategies. Final identification is again done by immuno-staining.

Density differential centrifugation can be used to separate CTCs and WBCs from RBCs using Ficoll-Paque (Pharmacia Fine Chemicals, Uppsala, Sweden) ^27^. This product can be directly used for molecular interrogation, flow cytometry, or further selection procedures.

The OncoQuick (Greiner Bio-One International GmbH, Germany) system precedes density centrifugation with size-based filtration to isolate CTCs ^28^, employing RT-PCR for detection. AccuCyte (RareCyte, Seattle, WA, USA) combines density centrifugation with immuno-staining and whole-exome sequencing to identify CTCs ^29^.

The unique electrical properties of CTCs are exploited by using a di-electrophoretic (DEP) field flow technology following a pre-CTC enrichment in the DEPArray (Menarini Silicon Biosystems, Castel Maggiore, Italy) ^30^ and ApoStream (ApoCell, Houston, TX, USA) instruments ^31, 32^.

Dr Huang’s lab at Pennsylvania State University demonstrated that an acoustic, on-chip flow cytometer (taSSAW) was able to separate CTCs from blood in flow ^33^. Acoustic separation exploits both size and compressibility differences to separate cell types ^34^.

## Analysis of isolated circulating tumor cells

CTCs show a wide range of phenotypic and genetic variations dependent on their primary tumor source. CTCs are, therefore, a very attractive source to use for differential diagnoses, prognoses, therapeutic target selections, and therapeutic response monitoring.

### Immunophenotyping of circulating tumor cells

Immunophenotyping of CTCs can reveal the biology of tumor origin for patients with “carcinoma of unknown primaries” (CUP). For example, multiplexed immunofluorescence staining of isolated CTCs with CK7, CK8, CK20, thyroid transcription factor 1 (TTF-1), estrogen receptor (ER), or prostate-specific antigen (PSA) could reveal the tissue of origin as the lung, differentiating it from breast, colorectal, and prostate cancer. CTCs provide a non-invasive diagnostic blood test as an adjunct to a tissue biopsy ^35^. Individual CTCs, CTC clusters, EMT-CTCs, and cancer-associated macrophage-like cells (CAMLs) all give support for metastatic disease. These observed variations and the immunophenotyping of CTCs have added prognostic value, e.g. the CTC cluster size and number are associated with lower overall survival in patients with breast, pancreatic, or prostate cancer ^36, 37^. CAMLs express epithelial, monocytic, and endothelial protein markers and represent engulfed CTCs. The significance of CAMLs to prognosis has not been defined; however, CAMLs may be evidence of early disease when identified in otherwise healthy individuals, thus serving as a screening tool ^23, 38^. The limitation of conventional immunophenotyping of CTCs is their rarity. In order to analyze multiple biomarkers in a single CTC, techniques have been developed using borohydride quenching on photoactivatable fluorophores of fluorescently stained CTCs, enabling “sequential re-staining” with additional biomarkers without the destruction of epitopes. Up to 10 markers have been shown to be feasible with re-stainings on a single CTC ^39^.

Immunohistochemical staining of matched primary tumor biopsies/cytology specimens shows striking morphological similarity between groups of cells within tumor and circulating tumor microemboli (CTM) from patients with non-small cell lung cancer (NSCLC) ^40^. Multiparameter flow cytometric and immunocytochemical analyses can detect, enumerate, and characterize CTCs ^41^. The detection of CTCs in whole blood using flow cytometry indicates metastatic tumor and has been used to monitor treatment effectiveness and disease prognosis ^42^. Multicolor flow cytometry allows detailed characterization by determining the expression of markers such as epidermal growth factor receptor (EGFR) and phosphorylated EGFR in CTCs ^43^.

Like solid tumor masses, CTCs are also heterogeneous. Capturing and examining both single CTCs and pools or clusters of CTCs can aid in the stratification of patients based on multiple parameters. Cell cycle staging could give another level of prognosis, and the identification of mitotic CTCs has been correlated with shortened overall survival ^44^.

Fluorescence *in situ* hybridization (FISH) is superior to protein assessment of HER-2 status in predicting response to HER-2-targeted immunotherapy in breast cancer patients, potentially identifying patients who may benefit from treatment adjustments ^45^. When FISH HER-2 on CTCs was compared to HER-2 status on the primary tumors, a 93% concordance in HER-2 status was observed ^46^.

### Genotyping of circulating tumor cells versus solid tumor

CTCs represent the dividing cells within the solid tumor mass, and single-cell sequencing has begun to unravel key questions in cancer invasion, metastasis, and therapy resistance that have been difficult to address with bulk tumor measurement ^47^. CTC mutations represent predictive and prognostic biomarkers and identify potential therapeutic targets.

Investigating whether the DNA mutational status of CTCs can represent that of the originating tumor is of great clinical importance. The mutational status of *KRAS*, *BRAF*, *CD133*, and Plastin3 ( *PLS3*) was probed in CTCs from patients with colorectal cancer and was compared with that of the originating tumor from the same patient. Discordance between the original tumor and CTCs for *KRAS*, *BRAF*, *CD133* rs3130, *CD133* rs2286455, and *PLS3* rs6643869 mutations was 5.77%, 3.85%, 11.54%, 7.69%, and 11.54%, respectively. This study supports the notion that the DNA mutational status of CTCs is a non-invasive, specific biomarker diagnostic tool ^48^.

CTCs enriched by CellSearch have been isolated by DEPArray to obtain single or pooled pure CTCs. Cell lysis yields pure tumor DNA and RNA. Whole-genome or whole-transcriptome amplification can be employed to analyze CTCs for tumor-specific mutations. CTCs of prostate cancer patients have been used to analyze somatic single-nucleotide variants.“ Census-based sequencing” combining multiple single CTC libraries markedly reduced the false-positive rate of somatic single-nucleotide variants ^49, 50^. NGS of CTC genomes has also identified distinct copy-number aberrations in patients with chemo-sensitive and chemo-refractory small-cell lung cancer (SCLC) ^51^. In additional studies, NGS analysis of single, pooled, and clusters of CTCs from six patients with metastatic breast cancer (MBC) revealed mutational heterogeneity in *PIK3CA*, *TP53*, *RB1*, and *ERBB2* genes, mutations matching those revealed in tissue NGS. In six single CTCs isolated from one MBC patient, one CTC had *TP53* R110 delC, one CTC had *TP53* R110 delG, and four single CTCs had the wild-type p53. Only a *TP53* R110 delC was obtained from 14 pooled CTCs isolated from the same patient. In the tumor breast tissue from the same patient, only *TP53* R110 delG mutation was detected. Single CTC and cluster analysis from another MBC patient showed mutational heterogeneity in *TP53*, *PIK3CA*, and two *ERBB2* mutations ^52– 54^. Such heterogeneity could be one explanation for the lack of response to targeted therapies.

### Additional functional analyses of circulating tumor cells

Viable CTCs would allow functional studies on the biology of CTCs, the identification of druggable therapeutic targets or pathways, and *in vitro* and *in vivo* drug testing. Analyses reported include direct single-cell lipido-metabolomics of CTCs from a neuroblastoma patient's blood analyzed with live single-cell mass spectrometry ^55^; in addition, microtubule bundling used as a marker of efficient taxane drug-target engagement has been visualized in CTCs isolated from castrate-resistant prostate cancer patients receiving taxane chemotherapy with docetaxel and paclitaxel, which has allowed the *ex vivo* examination of how sensitive CTCs are to taxane therapy. In one experiment, testing revealed an unperturbed microtubule network following docetaxel treatment; however, the addition of paclitaxel resulted in microtubule bundling, revealing a sensitivity to that drug ^56^, cell invasion assays using CTCs from ovarian cancer patients have been shown to be predictive of shorter disease-free survival ^57^, and epithelial immunospot (EPISPOT) assays have shown the capability of detecting proteins secreted/released/shed from single CTCs to distinguish between apoptotic and viable CTCs ^58^.

## Culture of circulating tumor cells

Culturing CTCs *in vitro*/ *in vivo* could facilitate drug development. Owing to the rarity of CTCs, it is necessary to culture CTCs and establish cell lines. Most isolated CTCs are not capable of dividing *in situ.* However, viable CTCs have been shown to proliferate in cell culture in response to stem cell growth factors such as epidermal growth factor (EGF) and fibroblast growth factor 2 (FGF2). The first permanent cell line came from CTCs of a patient with colon cancer: CTC-MCC-41 ^59^. CTCs isolated from 14 of 19 early stage lung cancer patients have been successfully expanded with a microfluidic *in situ* capture and grown in a three-dimensional co-culture model. These CTCs were verified to carry identical mutations from the primary tumors ^60^. Viable CTCs have also been isolated from the blood of a patient with CUP using a microfluidic flexible micro spring array (FMSA) device ^61^.

## Circulating tumor-cell-derived xenografts

The histological, transcriptomic, proteomic, and genomic similarities and comparable treatment responses between the originating tumors and patient-derived xenografts (PDXs) make PDXs useful models in research ^62^. CTC-derived xenografts (CDXs) are alternatives to PDXs when tumors are inaccessible or difficult to biopsy. CDXs from patients with either chemo-sensitive or chemo-refractory SCLC have been shown to recapitulate the drug-sensitivity patterns, genotyping, and clinical outcomes ^63^, and combinational treatment was shown to suppress tumor growth in a CDX from a chemo-refractory SCLC patient ^64^. Other CDX models have been cited.

## Comments and questions

What have we learned about CTCs? If a CTC or multiple CTCs are present then the cancer is metastatic by definition—it has traveled from its primary source. CTCs serve as a “silent” predictor of metastatic disease, and this is probably the most under-utilized but informative usage of CTCs overall. But all cancers are not equal in their ability to present as a CTC. In a disease-to-disease comparison when reviewing the percentage of patients exhibiting CTCs, CTCs are seen in a higher percentage of breast and prostate cancer patients than in colorectal cancer patients ^4^. Pancreatic ^65^ and ovarian cancers, regardless of their stage and aggressiveness, rarely reveal CTCs. The tissue source of a cancer can influence the likelihood of those cancerous cells entering the circulation. The physiologic and morphologic differences between various organs could predetermine a CTC’s ability to enter the circulation. In addition, some cells are more structurally “sound” than others and can withstand the forces in the circulatory system, while others will fragment. The vascularization within various tumors is not equal. The existence and type of lymphatic tissue, organized nodes versus diffuse lymphatic tissue found throughout the gastrointestinal tract or lungs, could greatly influence a tumor’s metastatic potential. These are all areas which require further investigation to uncover differences in CTC presence/absence in various cancers.

The continued research in the field of CTCs has shown these cells to be a valuable tumor source: “a liquid biopsy”. And because the body is always in a “cleaning mode” with the flux of lymphatic fluid over and through the tissues depositing waste into the blood circulation for disposal, that waste is the supplier of CTCs along with “cell-free circulating plasma”—also termed “a liquid biopsy”—and cell-free DNA (cfDNA) ^66– 68^. Do CTCs and cfDNA give the same information? No, cfDNA will present in all malignancies in both primary and metastatic disease once a detectable level is reached. However, cfDNA alone cannot predict progression from primary to metastatic disease. cfDNA can be used to detect minimal residual disease following initial treatment for curative intent (chemotherapy/surgery) and to detect recurrence ^69^. However, recurrence at the primary site or the spread to metastatic sites cannot be differentiated by cfDNA alone. An increase in cfDNA would be reflective of tumor burden but not where the tumor is, if it cannot be visualized on a scan.

Can CTCs and cfDNA be combined in such a manner to aid in the cure of metastatic disease? Yes, and used in combination and including a control of whole blood to detect somatic mutations, CTCs—the “silent” predictor of metastatic disease—and cfDNA can serve to monitor the progression of primary disease to a metastatic state and this combination can be used for every tumor type ^66– 71^. Collection algorithms for plasma and CTC determinations can be created with the first screening performed prior to any initial treatment. If CTCs are found (even a CTC level of 1), this is indicative of metastatic disease. Following curative treatment, cfDNA analysis should be employed to determine if there is residual disease and then continue cfDNA testing as surveillance. When increases are seen, the addition of CTCs to cfDNA can be made. cfDNA and CTCs will present significantly earlier than recurrent tumor visibility seen by CT/MRI scans. The advantages of CTCs and cfDNA include 1. they are a cost effective alternative to repeat negative scans, 2. they provide biological information to identify targetable mutations, and 3. they are superior to the routine cancer biomarkers currently followed ^70, 71^. Clinical trials need to be conducted to validate CTC/cfDNA evaluations following primary disease curative strategies with treatment induction again at the time of CTC/cfDNA emergence to determine overall survival elongations.

## References

[1] Ashworth T: A case of cancer in which cells similar to those in tumors were seen in the blood after death. *Australian Med J.* 1869;14:146.

[2] Alix-Panabières C Pantel K: Circulating tumor cells: liquid biopsy of cancer. *Clin Chem.* 2013;59(1):110–8. 10.1373/clinchem.2012.194258 23014601

[3] Gorges TM Tinhofer I Drosch M: Circulating tumour cells escape from EpCAM-based detection due to epithelial-to-mesenchymal transition. *BMC Cancer.* 2012;12:178. 10.1186/1471-2407-12-178 22591372 PMC3502112

[4] Allard WJ Matera J Miller MC: Tumor cells circulate in the peripheral blood of all major carcinomas but not in healthy subjects or patients with nonmalignant diseases. *Clin Cancer Res.* 2004;10(20):6897–904. 10.1158/1078-0432.CCR-04-0378 15501967

[5] Cristofanilli M Budd GT Ellis MJ: Circulating tumor cells, disease progression, and survival in metastatic breast cancer. *N Engl J Med.* 2004;351(8):781–91. 10.1056/NEJMoa040766 15317891

[6] de Bono JS Scher HI Montgomery RB: Circulating tumor cells predict survival benefit from treatment in metastatic castration-resistant prostate cancer. *Clin Cancer Res.* 2008;14(19):6302–9. 10.1158/1078-0432.CCR-08-0872 18829513

[7] Cohen SJ Alpaugh RK Gross S: Isolation and characterization of circulating tumor cells in patients with metastatic colorectal cancer. *Clin Colorectal Cancer.* 2006;6(2):125–32. 10.3816/CCC.2006.n.029 16945168

[8] Adams DL Stefansson S Haudenschild C: Cytometric characterization of circulating tumor cells captured by microfiltration and their correlation to the CellSearch ^®^ CTC test. *Cytometry A.* 2015;87(2):137–44. 10.1002/cyto.a.22613 25515318

[9] Li P Stratton ZS Dao M: Probing circulating tumor cells in microfluidics. *Lab Chip.* 2013;13(4):602–9. 10.1039/c2lc90148j 23306378 PMC3990734

[10] Harouaka R Kang Z Zheng SY: Circulating tumor cells: advances in isolation and analysis, and challenges for clinical applications. *Pharmacol Ther.* 2014;141(2):209–21. 10.1016/j.pharmthera.2013.10.004 24134902 PMC3947247

[11] Toss A Mu Z Fernandez S: CTC enumeration and characterization: moving toward personalized medicine. *Ann Transl Med.* 2014;2(11):108. 10.3978/j.issn.2305-5839.2014.09.06 25489582 PMC4245508

[12] Gorin MA Verdone JE van der Toom E: Circulating tumour cells as biomarkers of prostate, bladder, and kidney cancer. *Nat Rev Urol.* 2017;14(2):90–7. 10.1038/nrurol.2016.224 27872478

[13] Hayes DF Cristofanilli M Budd GT: Circulating tumor cells at each follow-up time point during therapy of metastatic breast cancer patients predict progression-free and overall survival. *Clin Cancer Res.* 2006;12(14 Pt 1):4218–24. 10.1158/1078-0432.CCR-05-2821 16857794

[14] Cohen SJ Punt CJ Iannotti N: Relationship of circulating tumor cells to tumor response, progression-free survival, and overall survival in patients with metastatic colorectal cancer. *J Clin Oncol.* 2008;26(19):3213–21. 10.1200/JCO.2007.15.8923 18591556

[15] Danila DC Heller G Gignac GA: Circulating tumor cell number and prognosis in progressive castration-resistant prostate cancer. *Clin Cancer Res.* 2007;13(23):7053–8. 10.1158/1078-0432.CCR-07-1506 18056182

[16] Nagrath S Sequist LV Maheswaran S: Isolation of rare circulating tumour cells in cancer patients by microchip technology. *Nature.* 2007;450(7173):1235–9. 10.1038/nature06385 18097410 PMC3090667

[17] Winer-Jones JP Vahidi B Arquilevich N: Circulating tumor cells: clinically relevant molecular access based on a novel CTC flow cell. *PLoS One.* 2014;9(1):e86717. 10.1371/journal.pone.0086717 24489774 PMC3906064

[18] Stott SL Hsu CH Tsukrov DI: Isolation of circulating tumor cells using a microvortex-generating herringbone-chip. *Proc Natl Acad Sci U S A.* 2010;107(43):18392–7. 10.1073/pnas.1012539107 20930119 PMC2972993

[19] Werner SL Graf RP Landers M: Analytical Validation and Capabilities of the Epic CTC Platform: Enrichment-Free Circulating Tumour Cell Detection and Characterization. *J Circ Biomark.* 2015;4 10.5772/60725 PMC5572988 28936239

[20] Andreopoulou E Urbauer D Krishnamurthy S: Comparison of circulating tumor cells (CTCs) in metastatic breast cancer (MBC): AdnaTest breast cancer for detection and biological characterization. *Clin Cancer Res.* 2008;14(19). Reference Source

[21] Saucedo-Zeni N Mewes S Niestroj R: A novel method for the *in vivo* isolation of circulating tumor cells from peripheral blood of cancer patients using a functionalized and structured medical wire. *Int J Oncol.* 2012;41(4):1241–50. 10.3892/ijo.2012.1557 22825490 PMC3583719

[22] Adams DL Zhu P Makarova OV: The systematic study of circulating tumor cell isolation using lithographic microfilters. *RSC Adv.* 2014;9:4334–42. 10.1039/C3RA46839A 25614802 PMC4299665

[23] Adams DL Martin SS Alpaugh RK: Circulating giant macrophages as a potential biomarker of solid tumors. *Proc Natl Acad Sci U S A.* 2014;111(9):3514–9. 10.1073/pnas.1320198111 24550495 PMC3948254

[24] Vona G Sabile A Louha M: Isolation by size of epithelial tumor cells : a new method for the immunomorphological and molecular characterization of circulatingtumor cells. *Am J Pathol.* 2000;156(1):57–63. 10.1016/S0002-9440(10)64706-2 10623654 PMC1868645

[25] Hvichia GE Parveen Z Wagner C: A novel microfluidic platform for size and deformability based separation and the subsequent molecular characterization of viable circulating tumor cells. *Int J Cancer.* 2016;138(12):2894–904. 10.1002/ijc.30007 26789903 PMC5069649

[26] Harouaka RA Zhou MD Yeh YT: Flexible micro spring array device for high-throughput enrichment of viable circulating tumor cells. *Clin Chem.* 2014;60(2):323–33. 10.1373/clinchem.2013.206805 24132944

[27] Weitz J Kienle P Lacroix J: Dissemination of tumor cells in patients undergoing surgery for colorectal cancer. *Clin Cancer Res.* 1998;4(2):343–8. 9516921

[28] Rosenberg R Gertler R Friederichs J: Comparison of two density gradient centrifugation systems for the enrichment of disseminated tumor cells in blood. *Cytometry.* 2002;49(4):150–8. 10.1002/cyto.10161 12454978

[29] Campton DE Ramirez AB Nordberg JJ: High-recovery visual identification and single-cell retrieval of circulating tumor cells for genomic analysis using a dual-technology platform integrated with automated immunofluorescence staining. *BMC Cancer.* 2015;15:360. 10.1186/s12885-015-1383-x 25944336 PMC4430903

[30] Fabbri F Carloni S Zoli W: Detection and recovery of circulating colon cancer cells using a dielectrophoresis-based device: *KRAS* mutation status in pure CTCs. *Cancer Lett.* 2013;335(1):225–31. 10.1016/j.canlet.2013.02.015 23419522

[31] Becker FF Wang X Huang Y: The removal of human leukaemia cells from blood using interdigitated microelectrodes. *J Phys D: Appl Phys.* 1994;27(12):2659–62. 10.1088/0022-3727/27/12/030

[32] Becker FF Wang XB Huang Y: Separation of human breast cancer cells from blood by differential dielectric affinity. *Proc Natl Acad Sci U S A.* 1995;92(3):860–4. 10.1073/pnas.92.3.860 7846067 PMC42720

[33] Li P Mao Z Peng Z: Acoustic separation of circulating tumor cells. *Proc Natl Acad Sci U S A.* 2015;112(16):4970–5. 10.1073/pnas.1504484112 25848039 PMC4413297

[34] Ding X Peng Z Lin SC: Cell separation using tilted-angle standing surface acoustic waves. *Proc Natl Acad Sci U S A.* 2014;111(36):12992–7. 10.1073/pnas.1413325111 25157150 PMC4246961

[35] Matthew EM Zhou L Yang Z: A multiplexed marker-based algorithm for diagnosis of carcinoma of unknown primary using circulating tumor cells. *Oncotarget.* 2016;7(4):3662–76. 10.18632/oncotarget.6657 26695546 PMC4826160

[36] Wang C Mu Z Chervoneva I: Longitudinally collected CTCs and CTC-clusters and clinical outcomes of metastatic breast cancer. *Breast Cancer Res Treat.* 2017;161(1):83–94. 10.1007/s10549-016-4026-2 27771841

[37] Fabisiewicz A Grzybowska E: CTC clusters in cancer progression and metastasis. *Med Oncol.* 2017;34(1):12. 10.1007/s12032-016-0875-0 28012133

[38] Adams DL Adams DK Alpaugh RK: Circulating Cancer-Associated Macrophage-Like Cells Differentiate Malignant Breast Cancer and Benign Breast Conditions. *Cancer Epidemiol Biomarkers Prev.* 2016;25(7):1037–42. 10.1158/1055-9965.EPI-15-1221 27197300 PMC4930681

[39] Adams DL Alpaugh RK Tsai S: Multi-Phenotypic subtyping of circulating tumor cells using sequential fluorescent quenching and restaining. *Sci Rep.* 2016;6: 33488. 10.1038/srep33488 27647345 PMC5028835

[40] Krebs MG Hou JM Sloane R: Analysis of circulating tumor cells in patients with non-small cell lung cancer using epithelial marker-dependent and -independent approaches. *J Thorac Oncol.* 2012;7(2):306–15. 22173704 10.1097/JTO.0b013e31823c5c16

[41] Racila E Euhus D Weiss AJ: Detection and characterization of carcinoma cells in the blood. *Proc Natl Acad Sci U S A.* 1998;95(8):4589–94. 10.1073/pnas.95.8.4589 9539782 PMC22534

[42] Hwu D Boutrus S Greiner C: Assessment of the role of circulating breast cancer cells in tumor formation and metastatic potential using *in vivo* flow cytometry. *J Biomed Opt.* 2011;16(4):40501. 10.1117/1.3560624 21529063

[43] Hristozova T Konschak R Budach V: A simple multicolor flow cytometry protocol for detection and molecular characterization of circulating tumor cells in epithelial cancers. *Cytometry A.* 2012;81(6):489–95. 10.1002/cyto.a.22041 22438318

[44] Adams DL Adams DK Stefansson S: Mitosis in circulating tumor cells stratifies highly aggressive breast carcinomas. *Breast Cancer Res.* 2016;18(1):44. 10.1186/s13058-016-0706-4 27142282 PMC4855427

[45] Frithiof H Aaltonen K Rydén L: A FISH-based method for assessment of *HER-2* amplification status in breast cancer circulating tumor cells following CellSearch isolation. *Onco Targets Ther.* 2016;9:7095–103. 10.2147/OTT.S118502 27895501 PMC5117892

[46] Mayer JA Pham T Wong KL: FISH-based determination of HER2 status in circulating tumor cells isolated with the microfluidic CEE™ platform. *Cancer Genet.* 2011;204(11):589–95. 10.1016/j.cancergen.2011.10.011 22200084

[47] Navin NE: The first five years of single-cell cancer genomics and beyond. *Genome Res.* 2015;25(10):1499–507. 10.1101/gr.191098.115 26430160 PMC4579335

[48] Lyberopoulou A Aravantinos G Efstathopoulos EP: Mutational analysis of circulating tumor cells from colorectal cancer patients and correlation with primary tumor tissue. *PLoS One.* 2015;10(4):e0123902. 10.1371/journal.pone.0123902 25902072 PMC4406761

[49] Speicher MR Pantel K: Tumor signatures in the blood. *Nat Biotechnol.* 2014;32(5):441–3. 10.1038/nbt.2897 24811515

[50] Lohr JG Adalsteinsson VA Cibulskis K: Whole-exome sequencing of circulating tumor cells provides a window into metastatic prostate cancer. *Nat Biotechnol.* 2014;32(5):479–84. 10.1038/nbt.2892 24752078 PMC4034575

[51] Carter L Rothwell DG Mesquita B: Molecular analysis of circulating tumor cells identifies distinct copy-number profiles in patients with chemosensitive and chemorefractory small-cell lung cancer. *Nat Med.* 2017;23(1):114–9. 10.1038/nm.4239 27869802

[52] Shaw JA Guttery DS Hills A: Mutation Analysis of Cell-Free DNA and Single Circulating Tumor Cells in Metastatic Breast Cancer Patients with High Circulating Tumor Cell Counts. *Clin Cancer Res.* 2017;23(1):88–96. 10.1158/1078-0432.CCR-16-0825 27334837 PMC6241844

[53] Fernandez SV Bingham C Fittipaldi P: *TP53* mutations detected in circulating tumor cells present in the blood of metastatic triple negative breast cancer patients. *Breast Cancer Res.* 2014;16(5):445. 10.1186/s13058-014-0445-3 25307991 PMC4303125

[54] Bingham C Fernandez SV Fittipaldi P: Mutational studies on single circulating tumor cells isolated from the blood of inflammatory breast cancer patients. *Breast Cancer Res Treat.* 2017;163(2):219–30. 10.1007/s10549-017-4176-x 28271309 PMC5410214

[55] Hiyama E Ali A Amer S: Direct Lipido-Metabolomics of Single Floating Cells for Analysis of Circulating Tumor Cells by Live Single-cell Mass Spectrometry. *Anal Sci.* 2015;31(12):1215–7. 10.2116/analsci.31.1215 26656808

[56] Kirby BJ Jodari M Loftus MS: Functional characterization of circulating tumor cells with a prostate-cancer-specific microfluidic device. *PLoS One.* 2012;7(4):e35976. 10.1371/journal.pone.0035976 22558290 PMC3338784

[57] Fan T Zhao Q Chen JJ: Clinical significance of circulating tumor cells detected by an invasion assay in peripheral blood of patients with ovarian cancer. *Gynecol Oncol.* 2009;112(1):185–91. 10.1016/j.ygyno.2008.09.021 18954898 PMC2606929

[58] Alix-Panabières C: EPISPOT assay: detection of viable DTCs/CTCs in solid tumor patients. *Recent Results Cancer Res.* 2012;195:69–76. 10.1007/978-3-642-28160-0_6 22527495

[59] Cayrefourcq L Mazard T Joosse S: Establishment and characterization of a cell line from human circulating colon cancer cells. *Cancer Res.* 2015;75(5):892–901. 10.1158/0008-5472.CAN-14-2613 25592149

[60] Zhang Z Shiratsuchi H Lin J: Expansion of CTCs from early stage lung cancer patients using a microfluidic co-culture model. *Oncotarget.* 2014;5(23):12383–97. 10.18632/oncotarget.2592 25474037 PMC4323004

[61] Gallant JN Matthew EM Cheng H: Predicting therapy response in live tumor cells isolated with the flexible micro spring array device. *Cell Cycle.* 2013;12(13):2132–43. 10.4161/cc.25165 23759587 PMC3737315

[62] Aparicio S Hidalgo M Kung AL: Examining the utility of patient-derived xenograft mouse models. *Nat Rev Cancer.* 2015;15(5):311–6. 10.1038/nrc3944 25907221

[63] Hodgkinson CL Morrow CJ Li Y: Tumorigenicity and genetic profiling of circulating tumor cells in small-cell lung cancer. *Nat Med.* 2014;20(8):897–903. 10.1038/nm.3600 24880617

[64] Potter DS Galvin M Brown S: Inhibition of PI3K/BMX Cell Survival Pathway Sensitizes to BH3 Mimetics in SCLC. *Mol Cancer Ther.* 2016;15(6):1248–60. 10.1158/1535-7163.MCT-15-0885 27197306

[65] Dotan E Alpaugh RK Ruth K: Prognostic Significance of MUC-1 in Circulating Tumor Cells in Patients With Metastatic Pancreatic Adenocarcinoma. *Pancreas.* 2016;45(8):1131–5. 10.1097/MPA.0000000000000619 26967453 PMC4983223

[66] Haber DA Velculescu VE: Blood-based analyses of cancer: circulating tumor cells and circulating tumor DNA. *Cancer Discov.* 2014;4(6):650–61. 10.1158/2159-8290.CD-13-1014 24801577 PMC4433544

[67] Ignatiadis M Lee M Jeffrey SS: Circulating Tumor Cells and Circulating Tumor DNA: Challenges and Opportunities on the Path to Clinical Utility. *Clin Cancer Res.* 2015;21(21):4786–800. 10.1158/1078-0432.CCR-14-1190 26527805

[68] Alix-Panabières C Pantel K: Clinical Applications of Circulating Tumor Cells and Circulating Tumor DNA as Liquid Biopsy. *Cancer Discov.* 2016;6(5):479–91. 10.1158/2159-8290.CD-15-1483 26969689

[69] Tie J Wang Y Tomasetti C: Circulating tumor DNA analysis detects minimal residual disease and predicts recurrence in patients with stage II colon cancer. *Sci Transl Med.* 2016;8(346):346ra92. 10.1126/scitranslmed.aaf6219 27384348 PMC5346159

[70] Danila DC Fleisher M Scher HI: Circulating tumor cells as biomarkers in prostate cancer. *Clin Cancer Res.* 2011;17(12):3903–12. 10.1158/1078-0432.CCR-10-2650 21680546 PMC3743247

[71] Tan CR Zhou L El-Deiry WS: Circulating Tumor Cells Versus Circulating Tumor DNA in Colorectal Cancer: Pros and Cons. *Curr Colorectal Cancer Rep.* 2016;12(3):151–61. 10.1007/s11888-016-0320-y 27516729 PMC4976692

